# Artificial Intelligence vs Clinician Performance in Estimating Probabilities of Diagnoses Before and After Testing

**DOI:** 10.1001/jamanetworkopen.2023.47075

**Published:** 2023-12-11

**Authors:** Adam Rodman, Thomas A. Buckley, Arjun K. Manrai, Daniel J. Morgan

**Affiliations:** 1 Department of Medicine, Beth Israel Deaconess Medical Center, Boston, Massachusetts; 2 Department of Computer Science, University of Massachusetts, Amherst, Massachusetts; 3 Department of Biomedical Informatics, Harvard Medical School, Boston, Massachusetts; 4 Department of Epidemiology and Public Health, University of Maryland School of Medicine, Baltimore; 5 Veterans Affairs Maryland Healthcare System, Baltimore

## Abstract

This diagnostic study compares the performance of artificial intelligence (AI) with that of human clinicians in estimating the probability of diagnoses before and after testing.

## Introduction

Diagnosis requires considering the likelihood of different diseases based on a patient’s presentation and updating those likelihoods based on diagnostic test results. However, practitioners often perform poorly at estimating the pretest and posttest probabilities of disease both in combining provided statistical information and in evaluating realistic patient scenarios.^1^ Large language models (LLMs) can convincingly solve difficult diagnostic cases, pass licensing examinations, and communicate empathetically with patients, suggesting that they have an emergent understanding of clinical reasoning.^2,3,4^ This diagnostic study assessed the ability of the artificial intelligence (AI) chatbot GPT-4 (OpenAI) to appropriately perform probabilistic reasoning by comparing its performance with a large survey of human clinicians.

## Methods

We used a previously published national survey, collected between June 1, 2018, and November 26, 2019, of 553 practitioners performing probabilistic reasoning in a series of 5 cases with scientific reference standards.^1^ Each case from the survey (eAppendixes 1 and 2 in Supplement 1) was copied into the model along with a prompt designed to make the AI commit to a specific pretest and posttest probability (eAppendix 3 in Supplement 1). Because LLMs are stochastic, an identical prompt was run in the LLM application programming interface 100 times at the default temperature (LLM setting to balance creative responses and consistency) of 1.0 on October 21, 2023, to develop a distribution of its outputs. Median (IQRs) of the LLM’s estimates were calculated, as were mean absolute error (MAE) and mean absolute percentage error (MAPE) for both LLM and human performance. The analysis and plotting were performed in R, version 4.3.0. This study was deemed exempt by the University of Maryland institutional review board because it was non–human participant research. The study followed the STROBE reporting guideline.

## Results

Compared with humans, the LLM had less error in pretest and posttest probability after a negative result in all 5 cases, even when the median LLM estimate differed more from the correct answer than the median human estimate; for example, for the asymptomatic bacteriuria case, the median pretest probability was 26% (IQR, 20%-30%) for the LLM vs 20% (IQR, 10%-50%) for humans and the MAE (MAPE) was 26.2 (5240%) vs 32.2 (6450%) (Table). Overall, the LLM had a narrower distribution of responses compared with humans, which likely explains this finding (Figure). The LLM was more accurate than humans in estimating posttest probability after a positive test result in 2 cases (breast cancer and hypothetical testing), similarly accurate in 2 cases, and less accurate in 1 case.

**Table.** Estimates of Probability of Disease Before Testing and After Positive or Negative Test Results for 5 Cases

Case	Reference probability, range, %	Estimated probability, median (IQR), %		MAE (MAPE)		Estimated probability, %		
		LLM (n = 100)	Clinicians (n = 553)	Clinicians	LLM	Residents (n = 290)	Attendings (n = 202)	APPs (n = 61)
**Pneumonia**								
Before test	25-42	72 (69-78)	80 (75-90)	47.3 (141)	39.5 (118)	80	85	80
After positive test result	46-65	95 (92-95)	95 (90-100)	38.4 (69)	38.5 (69)	95	95	95
After negative test result	10-19	25 (20-30)	50 (30-80)	39.5 (272)	10.7 (74)	60	50	50
**Breast cancer**								
Before test	0.2-0.3	1 (2-2)	5 (1-10)	8.3 (3350)	1.2 (466)	5	2	10
After positive test result	3-9	8 (7-8)	50 (30-80)	47.5 (791)	4.4 (74)	60	50	60
After negative test result	<.05	0.2 (0.06-0.3)	5 (1-10)	11.2 (45 000)	0.2 (751)	5	1	10
**Cardiac ischemia**								
Before test	1-4	3 (2-5)	10 (5-20)	11.8 (439)	1.3 (47)	10	5	15
After positive test result	2-11	69 (65-70)	70 (50-90)	56.2 (864)	56.5 (869)	75	60	90
After negative test result	0.4-3	5 (3-6)	5 (1-10)	8.6 (586)	4.0 (274)	5	5	10
**Asymptomatic bacteriuria (urinary tract infection)**								
Before test	0-1	26 (20-30)	20 (10-50)	32.2 (6450)	26.2 (5240)	25	20	30
After positive test result	0-8	90 (90-95)	80 (30-95)	62.2 (1500)	87.8 (2110)	78	90	90
After negative test result	0-0.1	5 (5-10)	5 (0-10)	11.8 (21 500)	7.3 (13 300)	5	5	5
**Hypothetical testing**								
After positive test result	2	2 (2-2)	95 (95-100)	74.4 (3720)	0.04 (2)	95	95	95
After negative test result	0	0 (0-0)	5 (0-5)	22.9 (∞)	0	2	5	5

Abbreviations: APPs, advanced practice practitioners; LLM, large language model; MAE, mean absolute error; MAPE, mean absolute percentage error.

**Figure.**
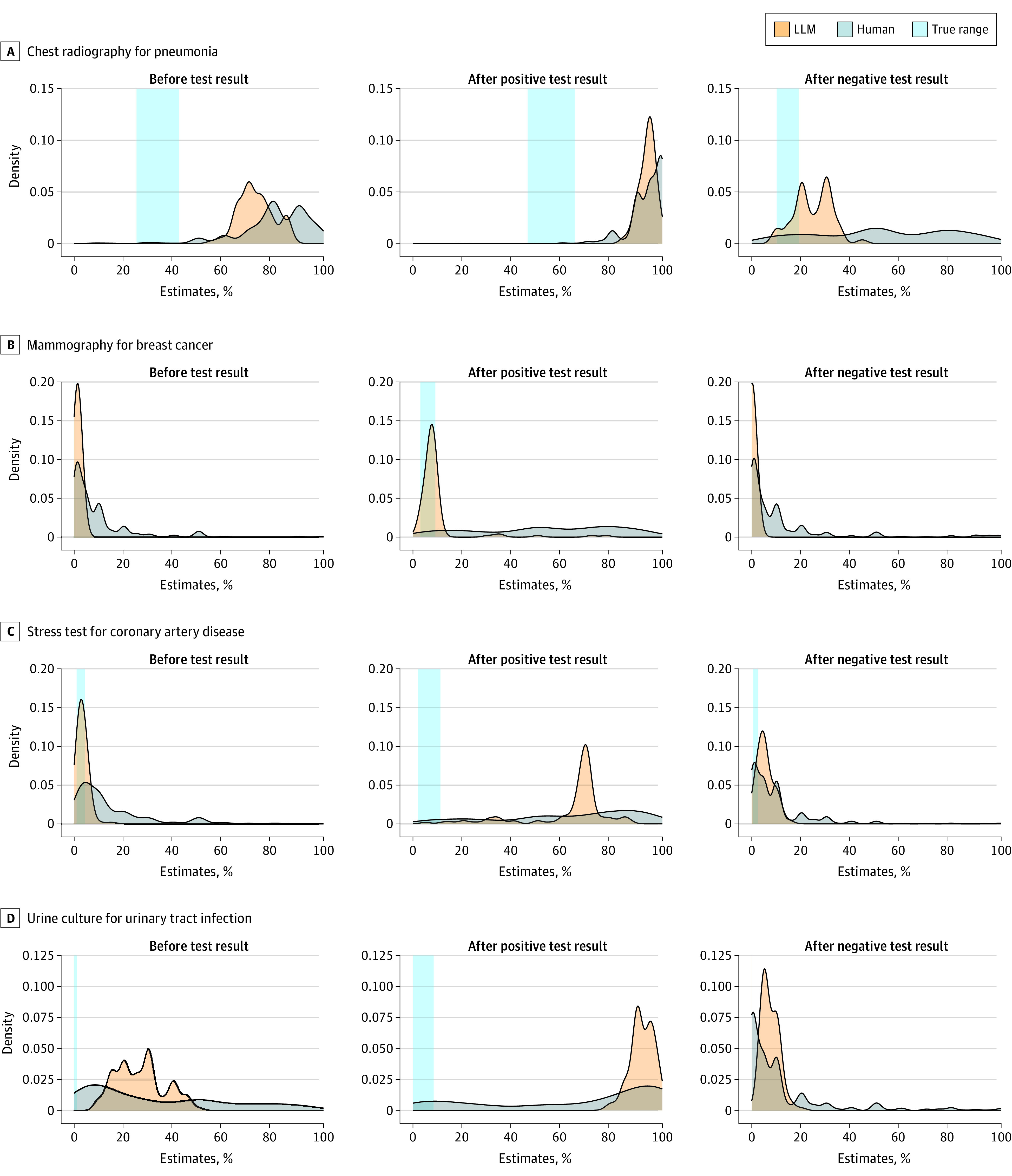
Density Plots of the Distributions of Responses LLM indicates large language model.

## Discussion

An LLM was more accurate than human clinicians in determining pretest and posttest probability after a negative test result in all 5 cases. The LLM did not perform as well after positive test results. LLM estimates were worse than human estimates for the case that was framed as a urinary tract infection (UTI) in the question stem but was actually asymptomatic bacteriuria; some human clinicians recognized this, but the model did not and likely gave estimates assuming the diagnosis of UTI was accurate. Other than the fifth test case, when the AI formally solved a basic statistical reasoning question, the range of the LLM’s probability outputs in response to clinical vignettes appeared to be emergent from its stochastic nature.

Study limitations include use of a simple input-output prompt design strategy; other approaches may yield better results and deserve investigation. Cases were also simplistic in order to have clear reference standards. Future research will need to investigate LLM performance in more complex cases.

It is not clear why performance of the LLM was not as strong in posttest probability after a positive result. However, even if imperfect, probabilistic recommendations from LLMs might improve human diagnostic performance through collective intelligence, especially if AI diagnostic aids can combine probabilistic, narrative, and heuristic approaches to diagnosis.^5^

## Notes

Supplement 1. **eAppendix 1.** Survey Questions **eAppendix 2.** Best Answers for Testing Questions **eAppendix 3.** GPT-4 Prompts

Supplement 2.
Data Sharing Statement
