# Supplementary material for: Artificial Intelligence vs Clinician Performance in Estimating Probabilities of Diagnoses Before and After Testing
Source: JAMA Netw Open. 2023 Dec 11;6(12):e2347075. doi: 10.1001/jamanetworkopen.2023.47075 (PMC10714249; doi:10.1001/jamanetworkopen.2023.47075)
Supplement: Supplement 2. — Data Sharing Statement [file jamanetwopen-e2347075-s002.pdf]

Data Sharing Statement

Rodman. Artificial Intelligence vs Clinician Performance in Estimating Probabilities of Diagnoses Before and After Testing. *JAMA Netw Open*. Published December 11, 2023. doi:10.1001/jamanetworkopen.2023.47075

Data
Data available: Yes
Data types: Data (not involving human participants)
How to access data: Data is available from the first author on request.
When available: With publication

Supporting Documents
Document types: None

Additional Information
Who can access the data: Data will be made available to anyone requesting it.
Types of analyses: Data will be made available for any purpose.
Mechanisms of data availability: Available to anyone.
